# Supplementary material for: ARMCX3 Mediates Susceptibility to Hepatic Tumorigenesis Promoted by Dietary Lipotoxicity
Source: Cancers (Basel). 2021 Mar 5;13(5):1110. doi: 10.3390/cancers13051110 (PMC7961652; doi:10.3390/cancers13051110)
Supplement: Supplementary file 1 [file cancers-13-01110-s001.zip › supplementary material/cancers-1127769--suppl.docx]

Supplementary Materials: ARMCX3 Mediates Susceptibility to Hepatic Tumorigenesis Promoted by Dietary Lipotoxicity

Serena Mirra, Aleix Gavaldà-Navarro, Yasmina Manso, Mónica Higuera, Román Serrat, María Teresa Salcedo,
Ferran Burgaya, José Maria Balibrea, Eva Santamaría, Iker Uriarte, Carmen Berasain, Matias A. Avila,
Beatriz Mínguez, Eduardo Soriano and Francesc Villarroya

**Table S1.** Clinical characteristics of 24 patients of bariatric surgery used in the study.

| **Demographics** | | |
| --- | --- | --- |
| Gender, *n* (%) | Male | 45.8% |
| Age, median (range) |  | 43.69 (58–20) |
| Race/Ethnicity, *n* (%) | Caucasian | 70.8% |
|  | Hispanic | 29.2% |
| **Histology** | | |
| NASH CRN | 1/8 | 16.6% |
|  | 2/8 | 8.33% |
|  | 3/8 | 12.5% |
|  | 4/8 | 33.33% |
|  | 5/8 | 4.16% |
|  | 6/8 | 4.16% |
|  | 7/8 | 8.33% |
|  | 8/8 | 4.16% |
| Fibrosis | F0 | 15% |
|  | F1 | 48% |
|  | F2 | 37% |
| **Laboratory values** | | |
| Bilirubin mg/dL, median (range) |  | 0.65 (0.69−0.61) |
| Albumin g/L, median (range) |  | 3.86 (4.34−2.9) |
| Diabetes |  | 16.6% |
| Dyslipidemia |  | 29.2% |
| Hypertension |  | 41.7% |

**Table S2.** Clinical characteristics of the 19 control patients.

| **Demographics.** | | |
| --- | --- | --- |
| Gender, *n* (%) | Male | 85% |
| Age, median (range) |  | 62.9 (76–47) |
| Race/Ethnicity, *n* (%) | Caucasian | 92% |
|  | Hispanic | 8% |
| **Laboratory values** | | |
| Bilirubin mg/dL, median (range) |  | 0.43 (0.71–0.28) |
| Albumin g/L, median (range) |  | 3 (4.4–2.3) |
| Diabetes |  | 26% |
| Dyslipidemia |  | 8% |
| Hypertension |  | 23% |

**Table S3.** Liver function tests in fArmcx3/Cre- (Control) and ARMCX3-KO (KO) mice fed low-fat diet (LFD) or high-fat diet (HFD) for 16 weeks. ALP, alkaline phosphatase; AST, aspartate aminotransferase; LDH, lactate dehydrogenase. Mean ± SEM (*n* = 6–8).

| **header** | **Control LFD** | **Control HFD** | **KO LFD** | **KO HFD** | **ANOVA Control v. KO** | **ANOVA LFD v. HFD** |
| --- | --- | --- | --- | --- | --- | --- |
| **ALP activity (U/L)** | 11.1 ± 1.6 | 13.0 ± 1.7 | 9.9 ± 0.5 | 9.6 ± 0.8 | *p* = 0.19 | *p* = 0.64 |
| **AST activity (U/L)** | 183 ± 41 | 232 ± 23 | 190 ± 37 | 175 ± 26 | *p* = 0.50 | *p* = 0.64 |
| **LDH activity (U/L)** | 914 ± 232 | 1093 ± 205 | 820 ± 74 | 897 ± 86 | *p* = 0.52 | *p* = 0.57 |

**Table S4.** Liver function tests in fArmcx3/Cre- (Control) and ARMCX3-KO (KO) mice treated with DEN and fed low-fat diet (LFD) or high-fat diet (HFD) for 24 weeks. ALP, alkaline phosphatase; AST, aspartate aminotransferase; LDH, lactate dehydrogenase. Mean ± SEM (*n* = 9–13).

| **header** | **Control LFD** | **Control HFD** | **KO LFD** | **KO HFD** | **ANOVA Control v. KO** | **ANOVA LFD v. HFD** |
| --- | --- | --- | --- | --- | --- | --- |
| **ALP activity  (U/L)** | 12.3 ± 1.1 | 12.8 ± 3.5 | 10.7 ± 0.8 | 10.9 ± 0.8 | *p* = 0.39 | *p* = 0.85 |
| **AST activity  (U/L)** | 189 ± 13 | 262 ± 29 | 202 ± 24 | 267 ± 41 | *p* = 0.75 | *p* = 0.02 |
| **LDH activity (U/L)** | 897 ± 95 | 903 ± 151 | 664 ± 93 | 1016 ± 141 | *p* = 0.62 | *p* = 0.15 |


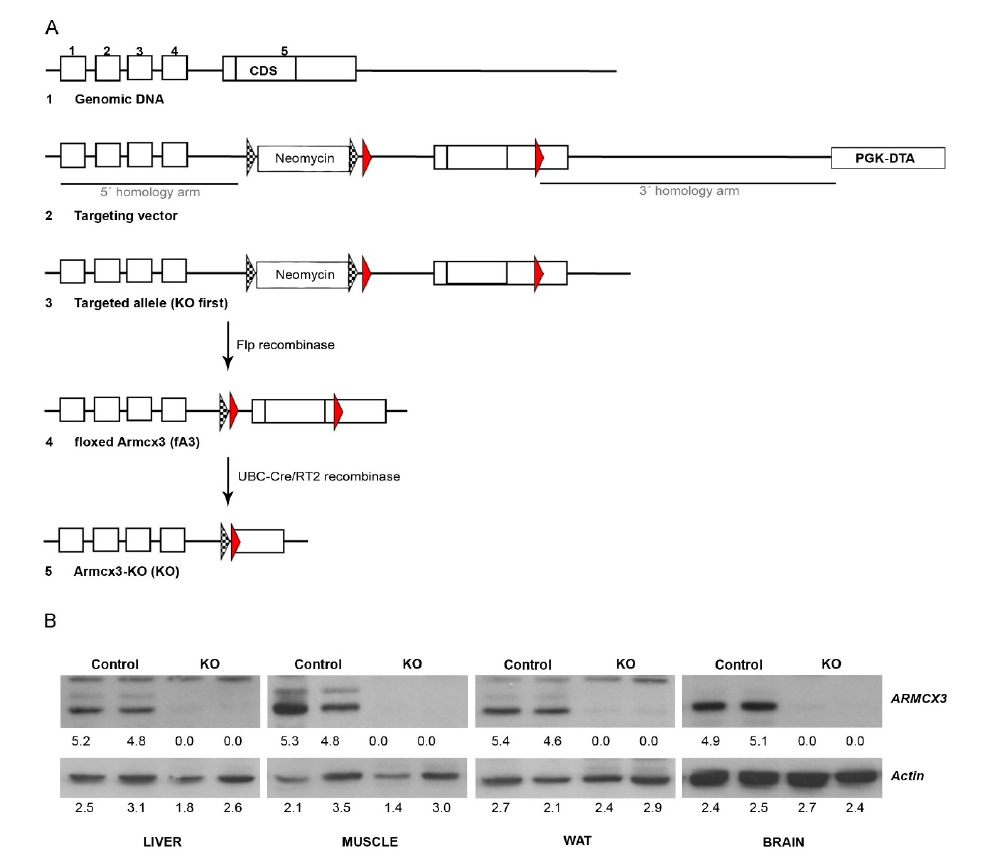


**Figure S1.**


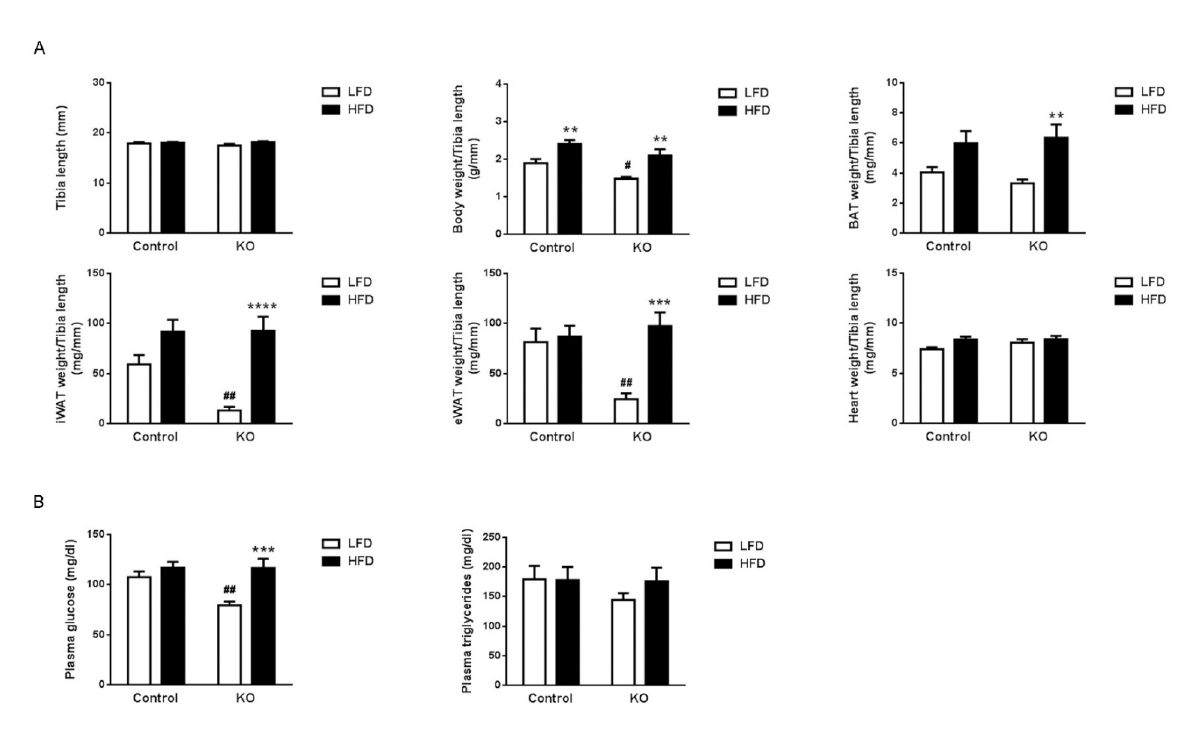


**Figure S2.**


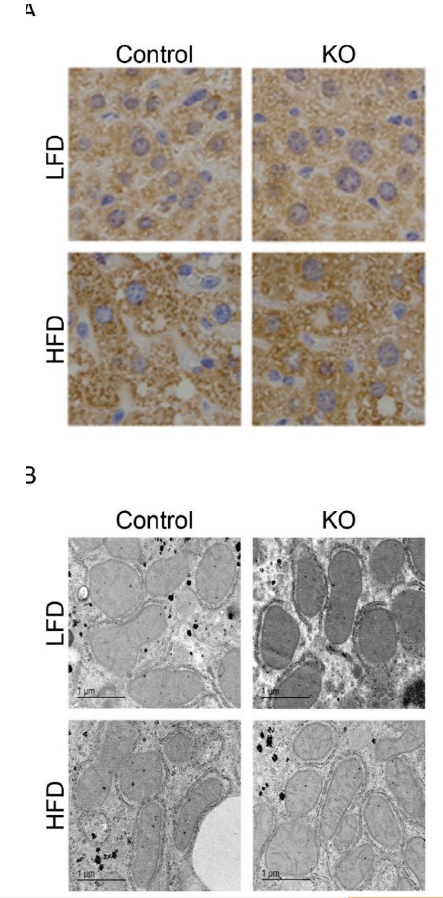


**Figure S3.**


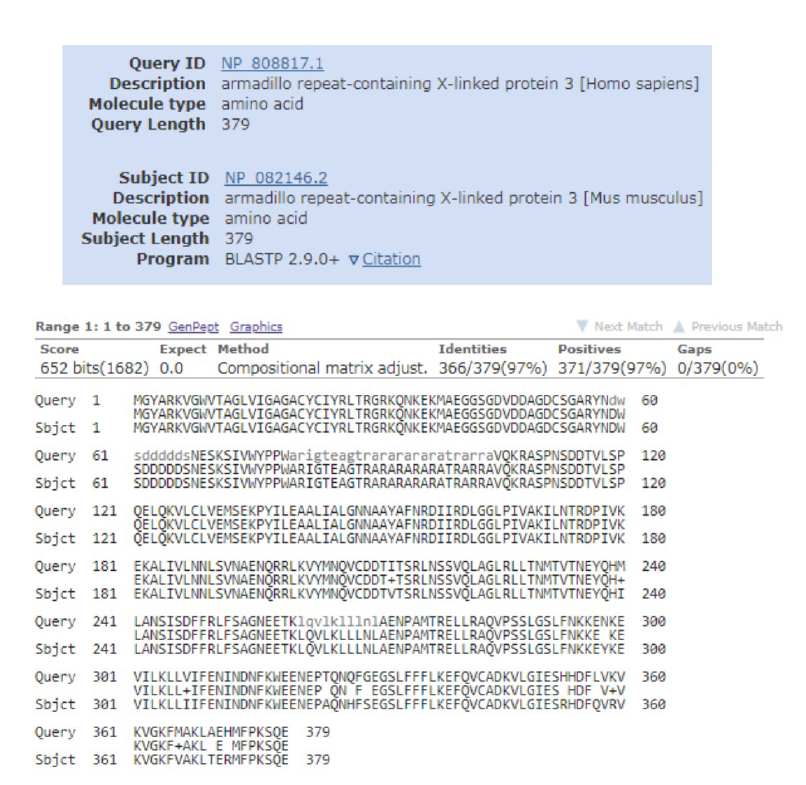


**Figure S4.**


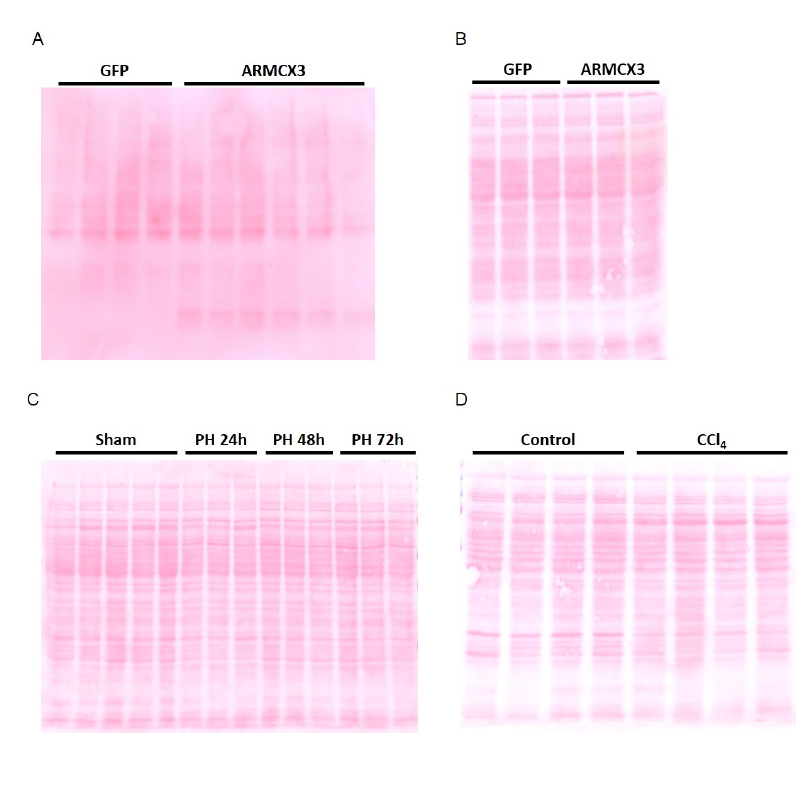


**Figure S5.**


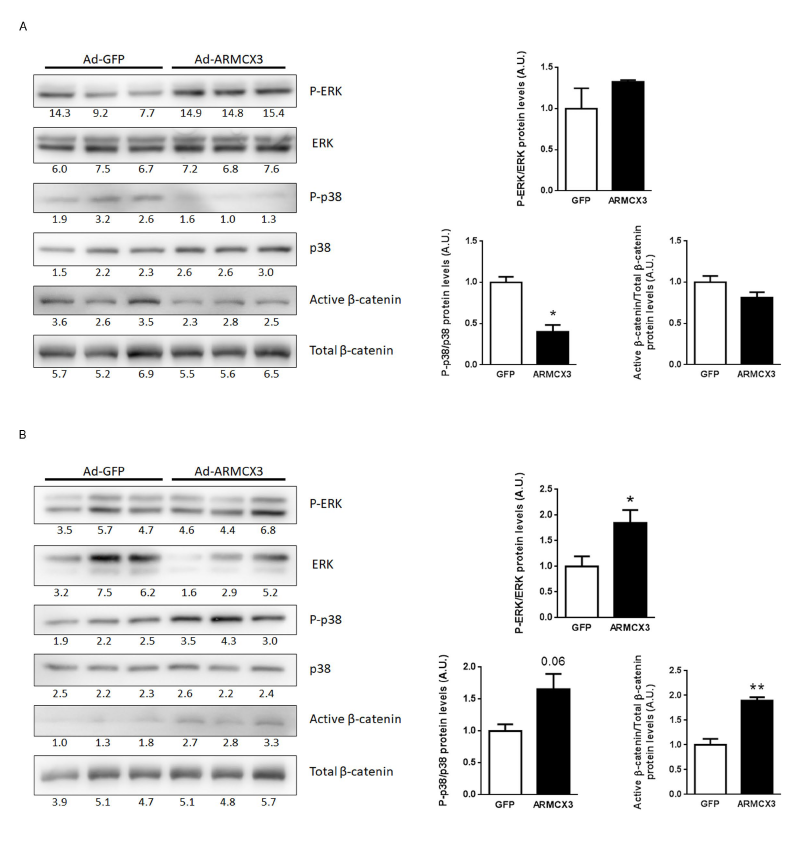


**Figure S6.**
