# Supplementary material for: ARMCX3 Mediates Susceptibility to Hepatic Tumorigenesis Promoted by Dietary Lipotoxicity
Source: Cancers (Basel). 2021 Mar 5;13(5):1110. doi: 10.3390/cancers13051110 (PMC7961652; doi:10.3390/cancers13051110)
Supplement: Supplementary file 1 [file cancers-13-01110-s001.zip › supplementary material/File S1. The original uncropped image.pdf]

Full unedited gel for Figure 1A

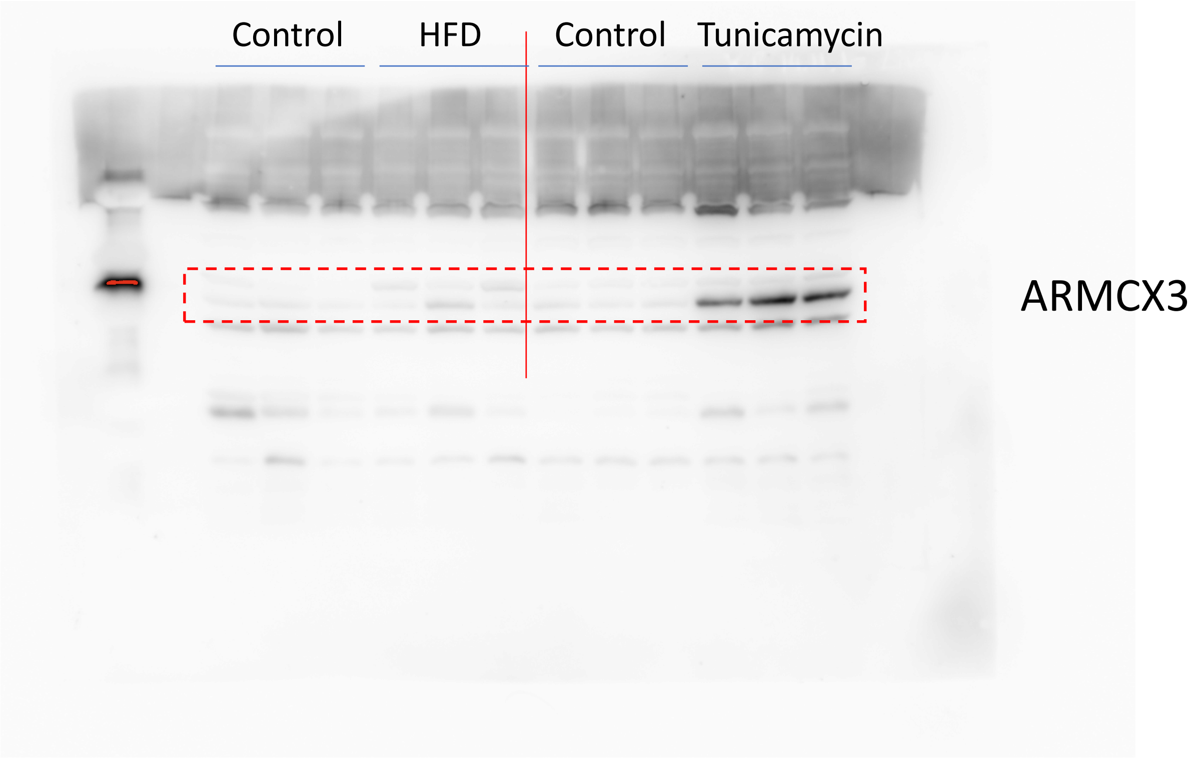

Ponceau's staining

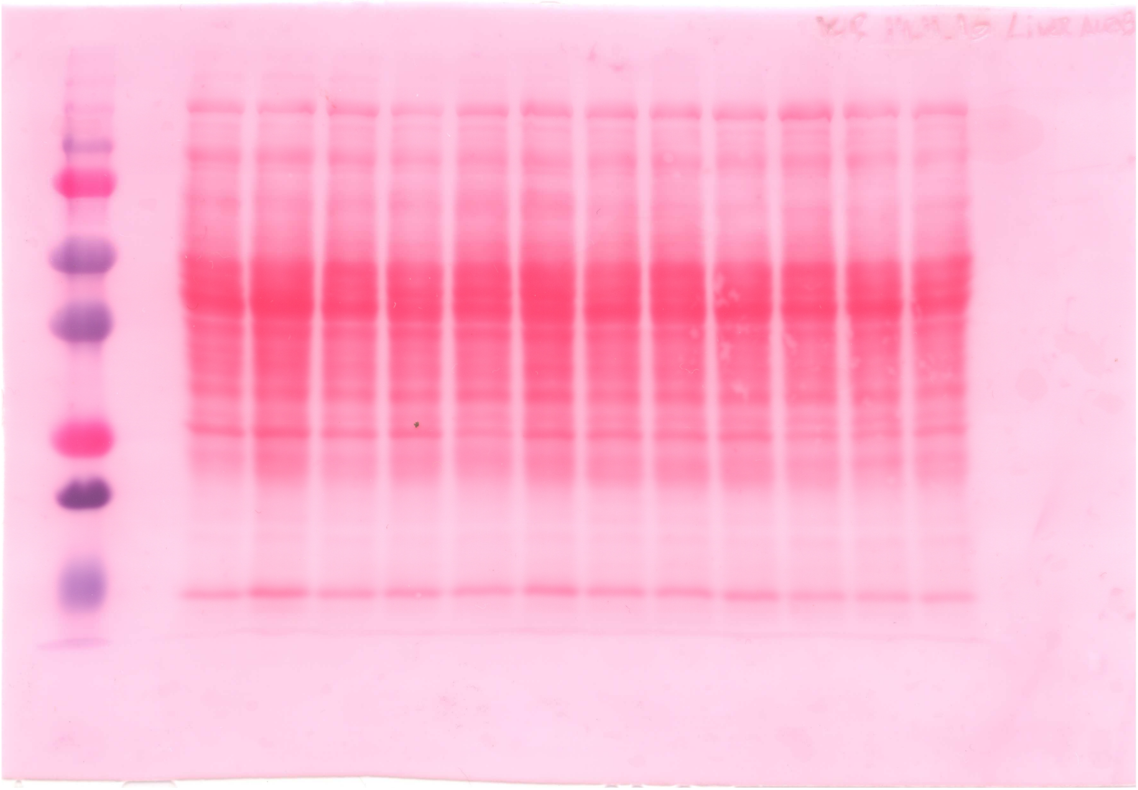

Full unedited gel for Figure 4B Left

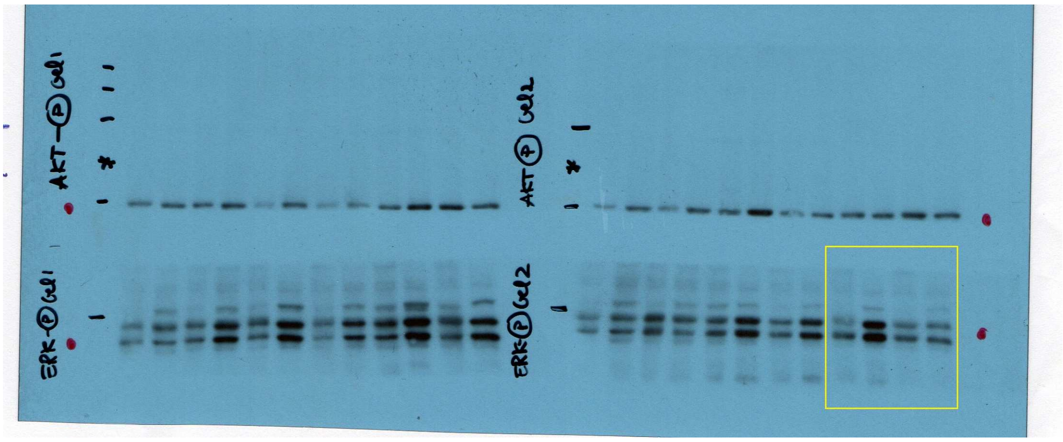

P-ERK

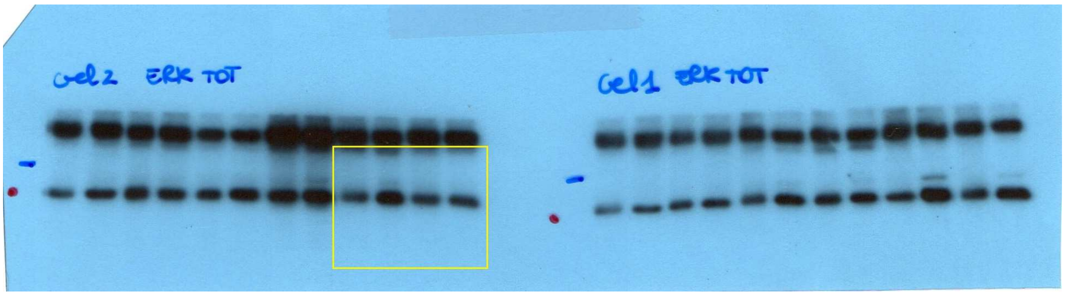

Total ERK

Ponceau's staining

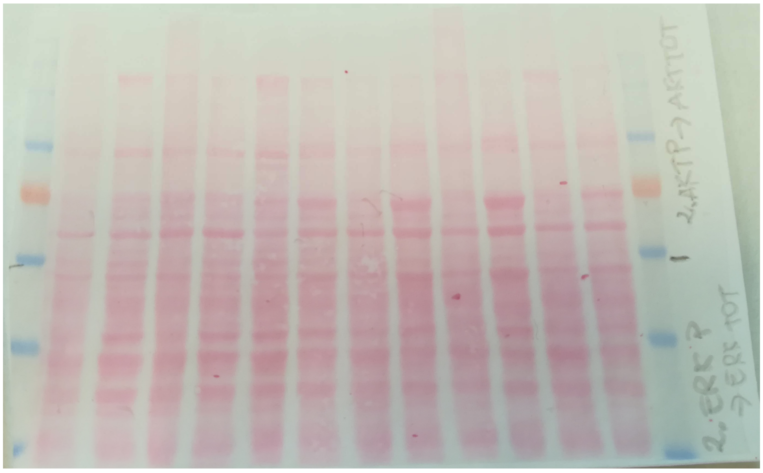

Full unedited gel for Figure 4B Right

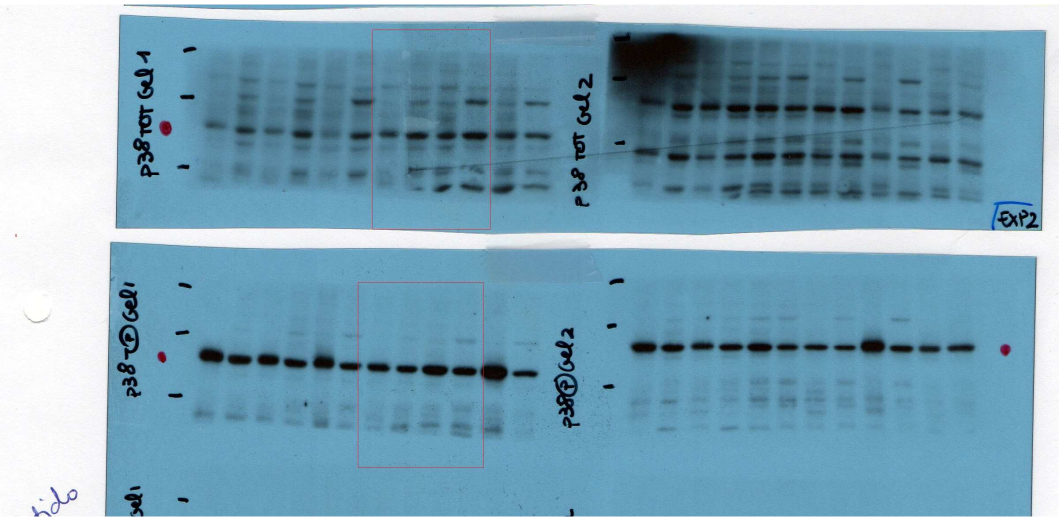

p38

P-p38

Ponceau's staining

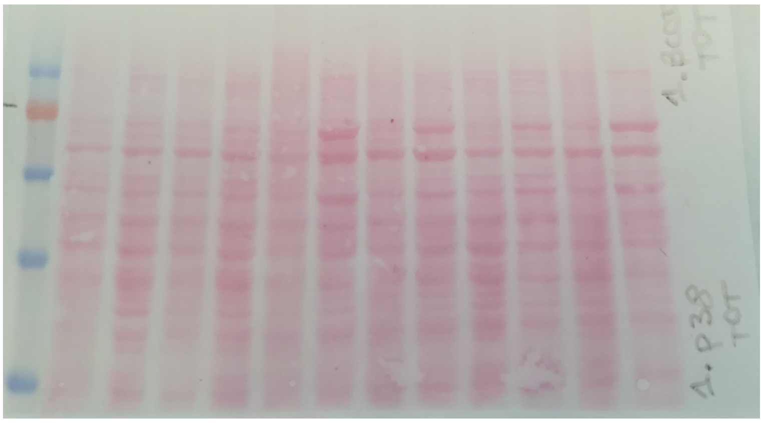

p38 blot

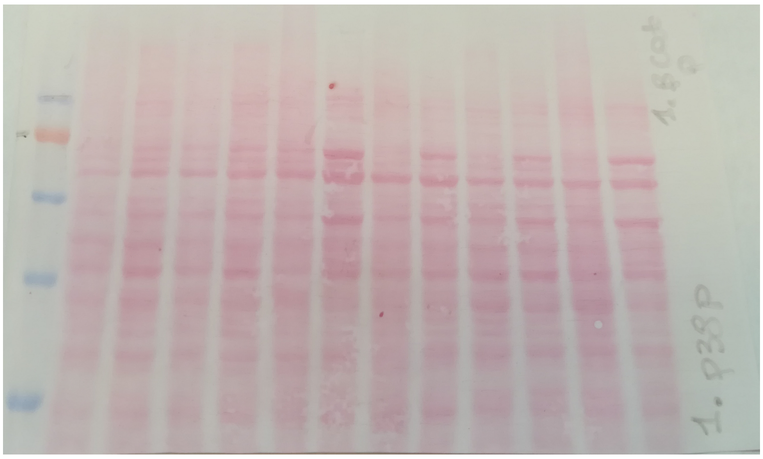

P-p38 blot

Full unedited gel for Figure 6B

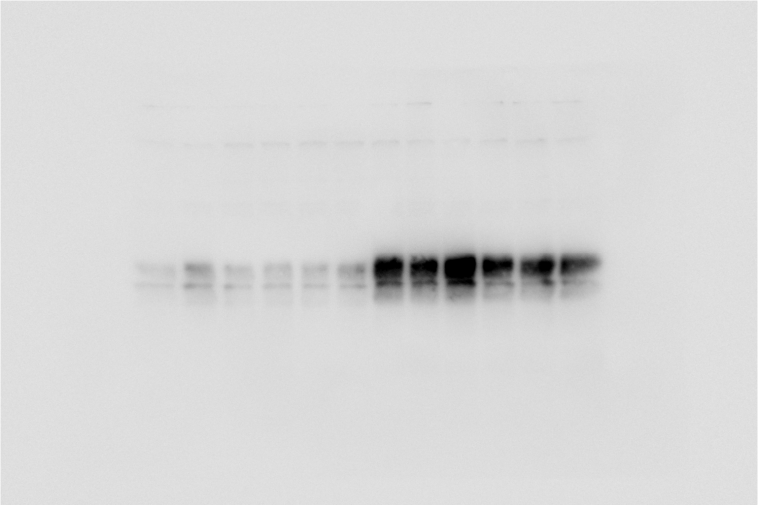

ARMCX3

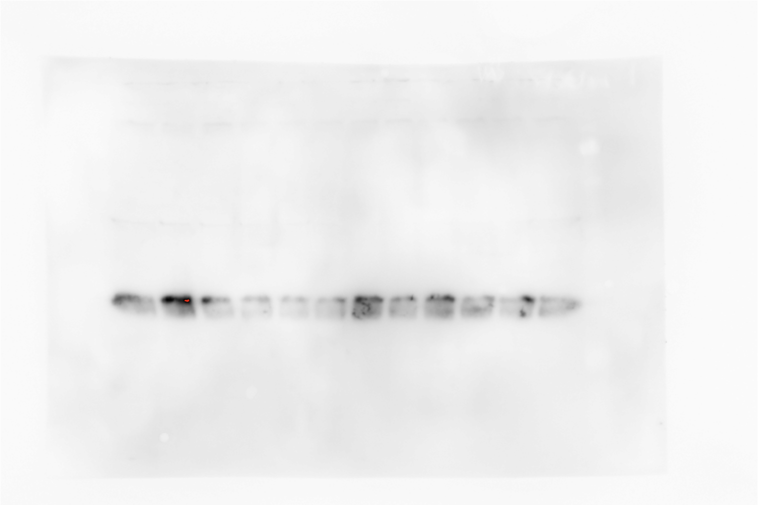

PCNA

Ponceau's staining

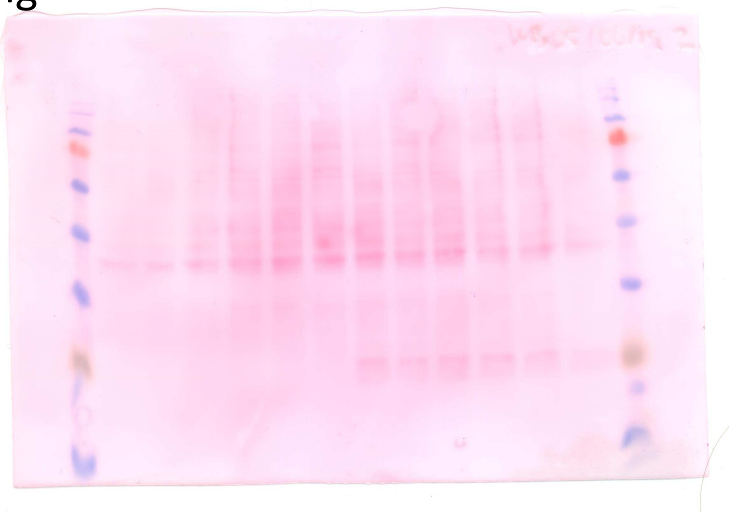

Full unedited gel for Figure 6C

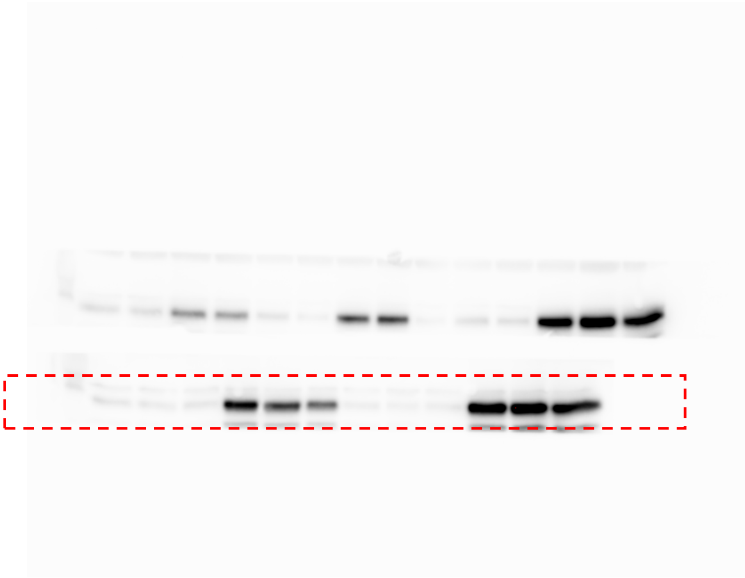

ARMCX3

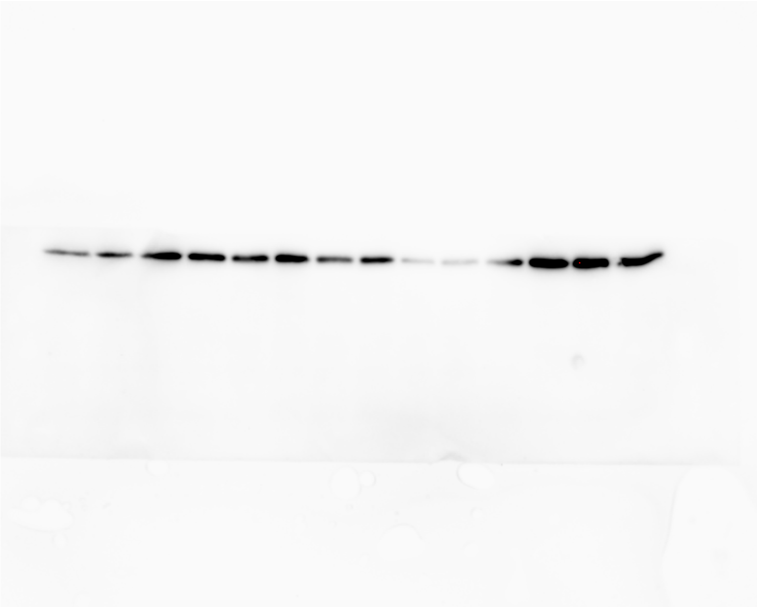

PCNA

Ponceau's staining

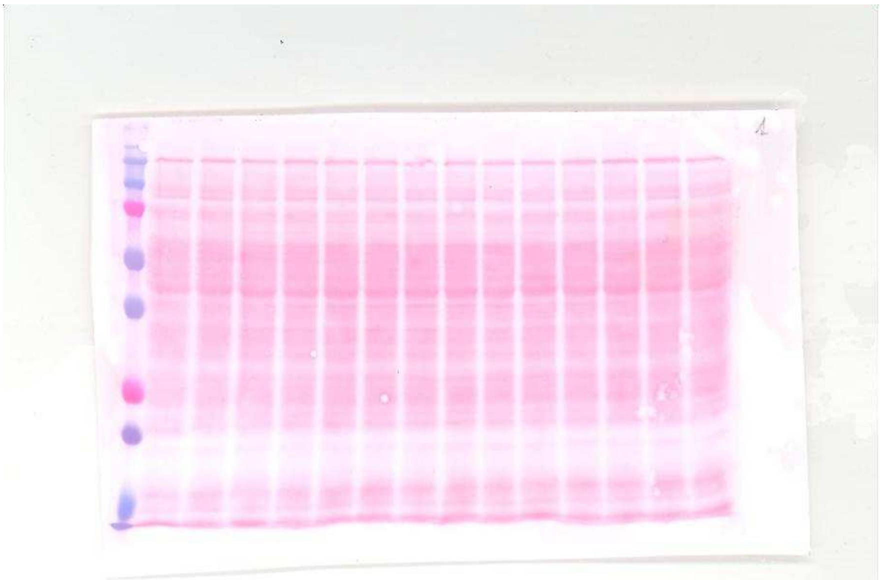

Full unedited gel for Figure 6D Left

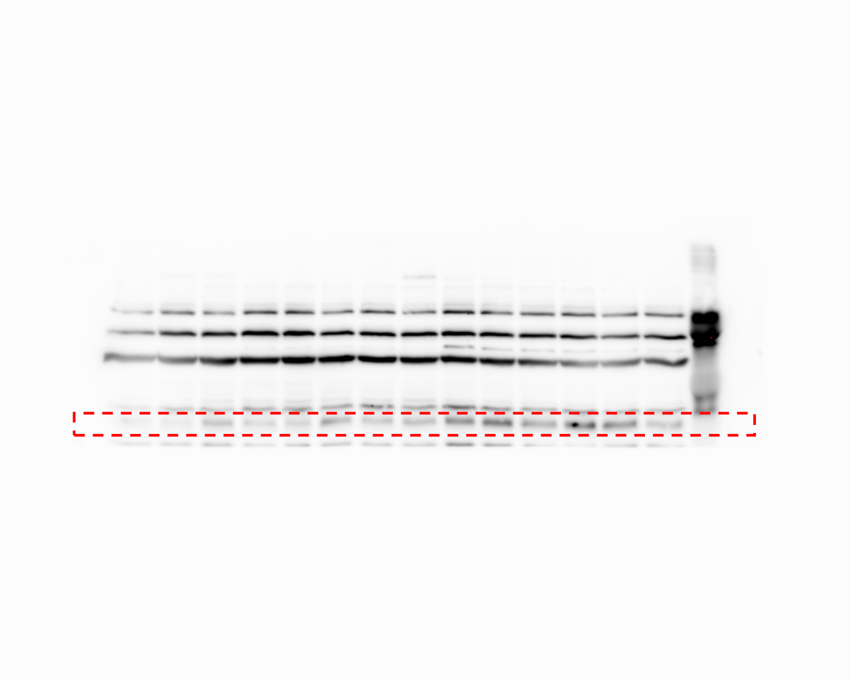

ARMCX3

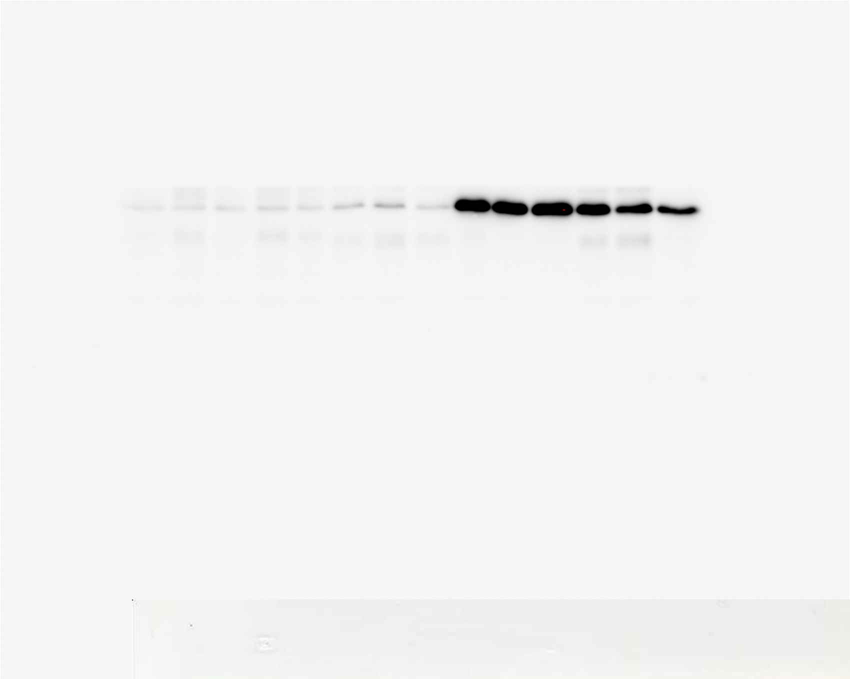

PCNA

Ponceau’s staining

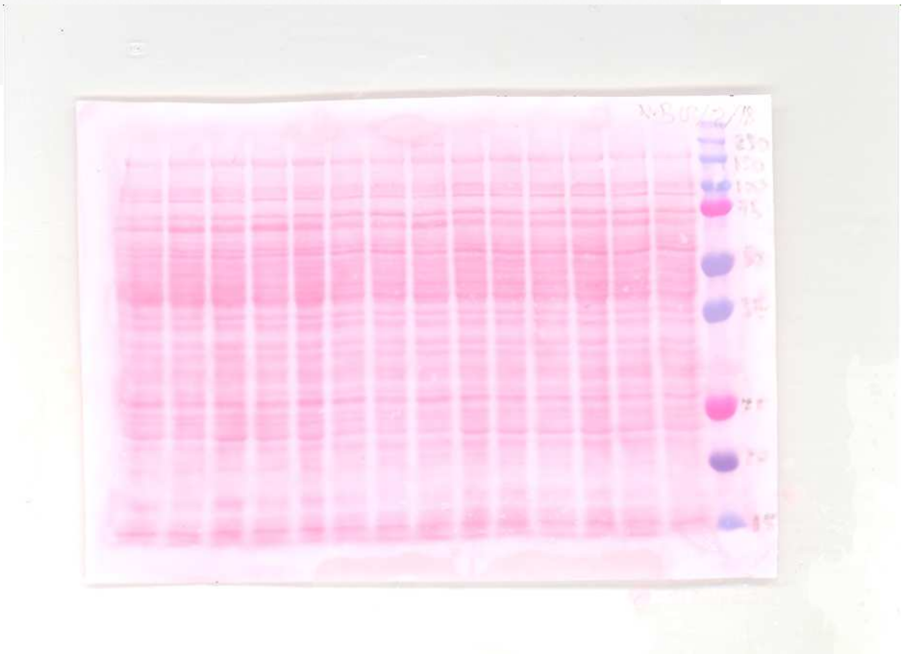

Full unedited gel for Figure 6D Right

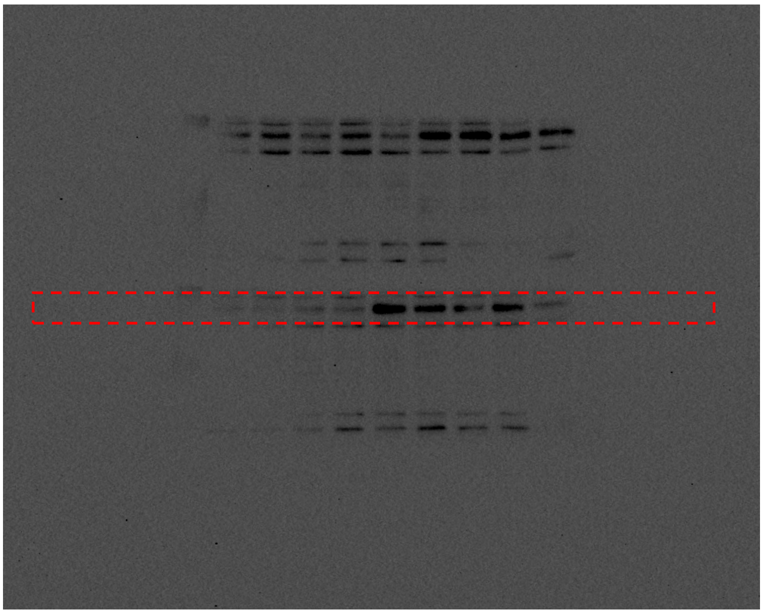

ARMCX3

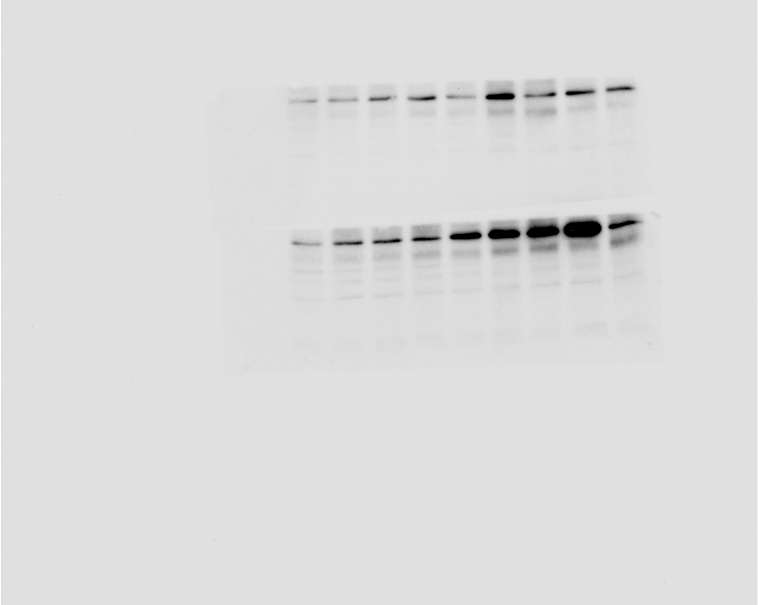

PCNA

Ponceau’s staining

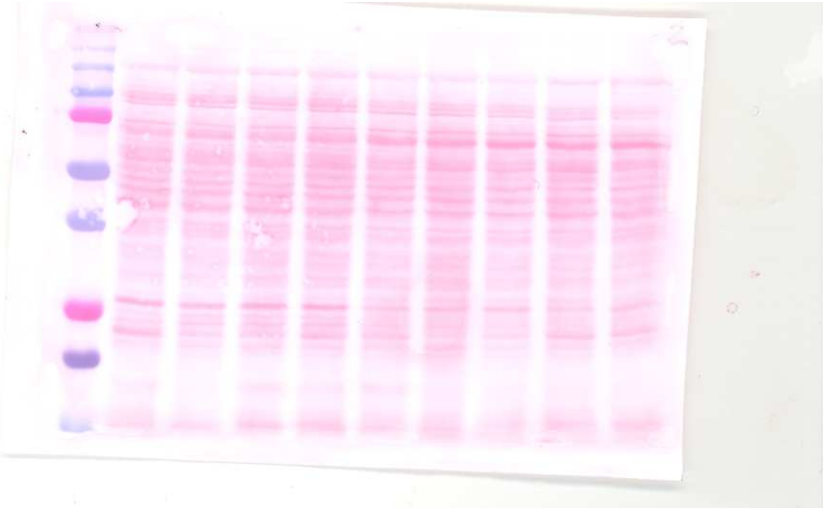

Full unedited gel for Figure 7A

Same membrane cut in 2 pieces for each antibody incubation

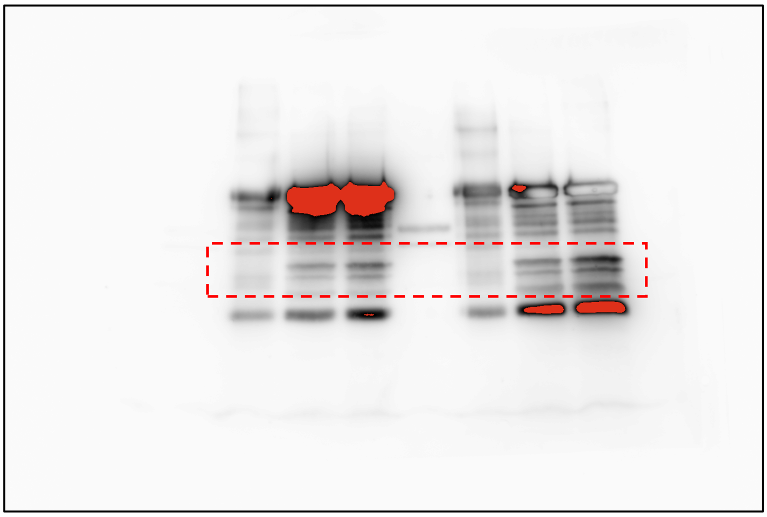

ARMCX3

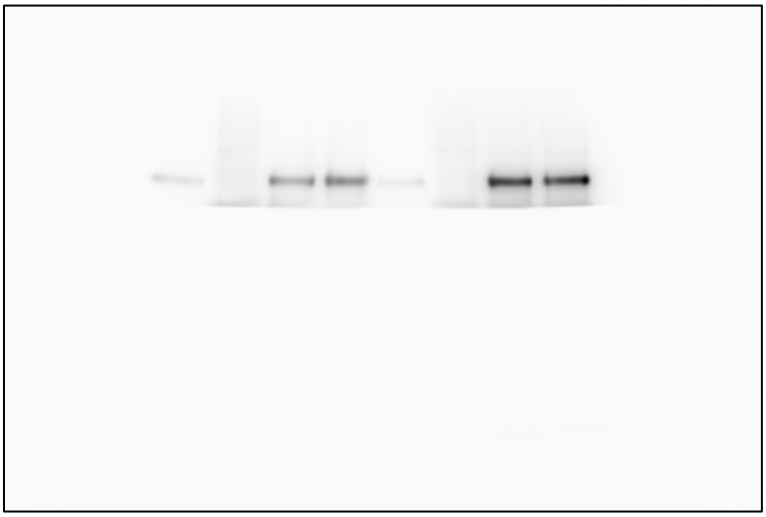

SOX9

Full unedited gel for Figure7B

Same membrane cut in 3 pieces for each antibody incubation

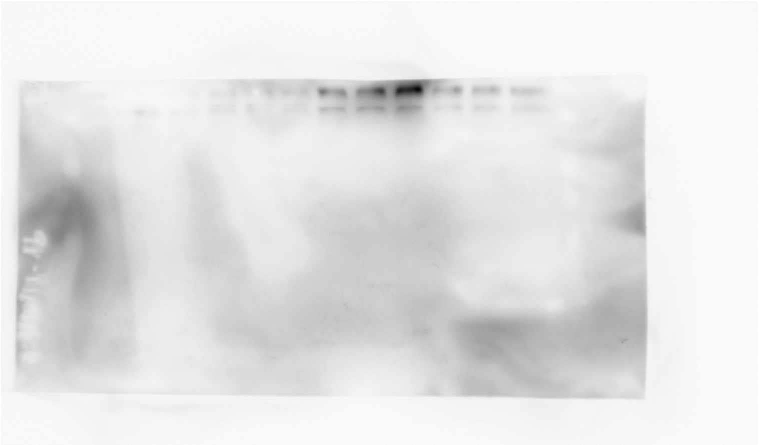

ARMCX3

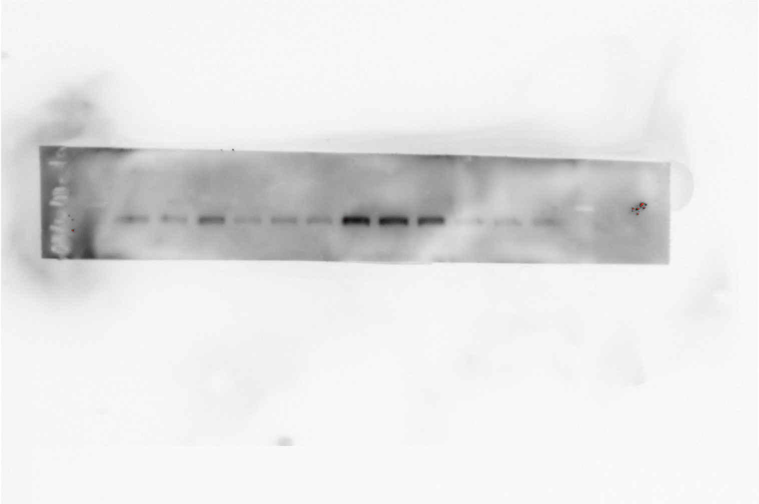

SOX9

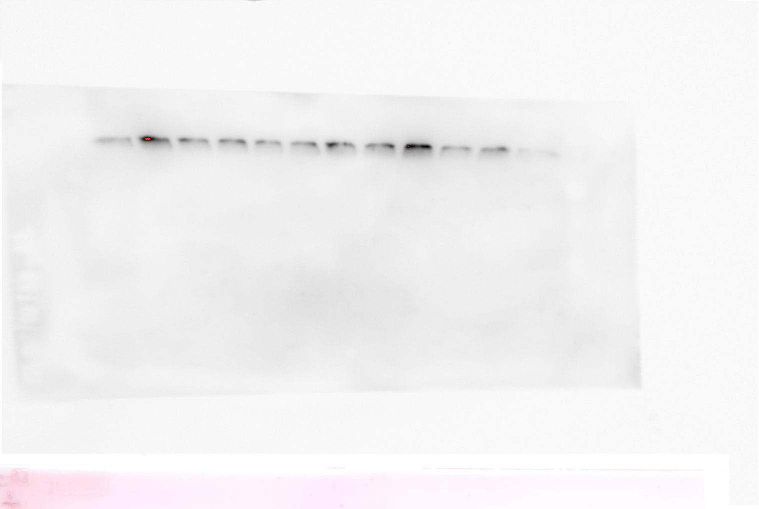

PCNA

Ponceau’s staining

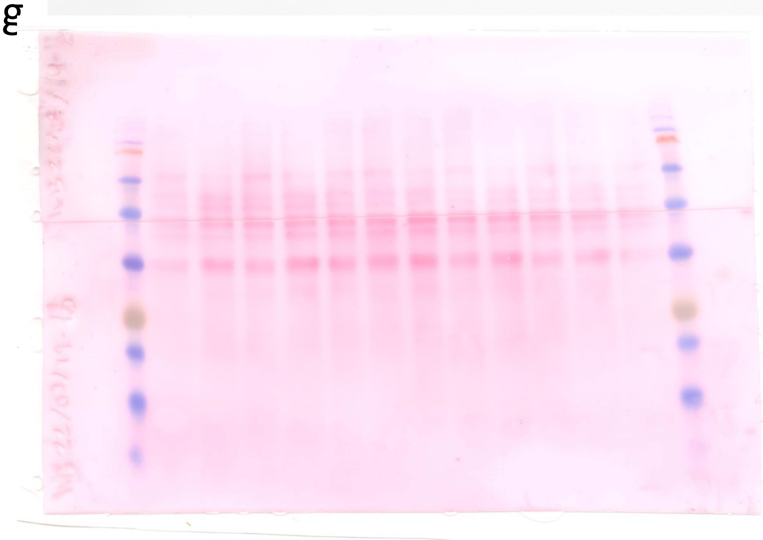

Full unedited gel for Figure7C

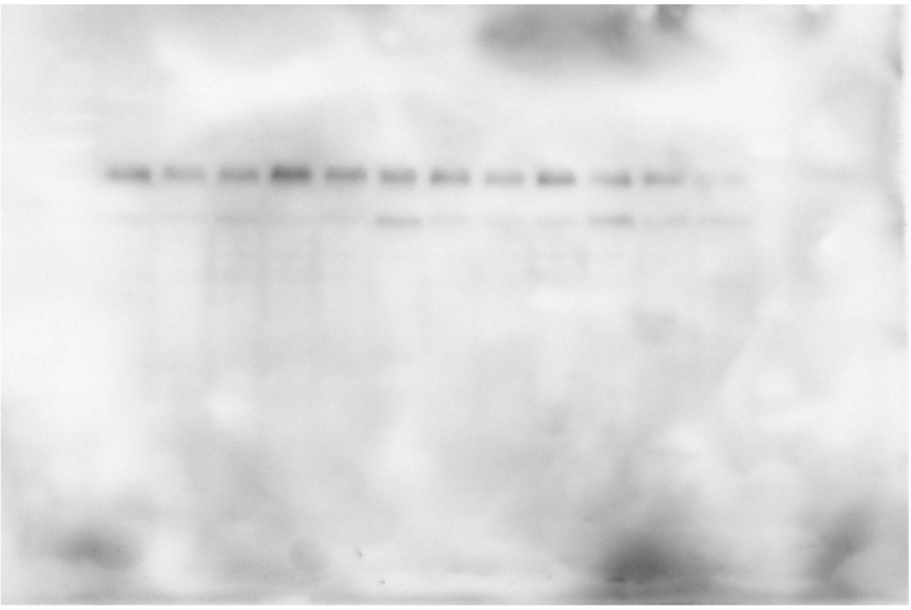

SOX9

Ponceau's staining

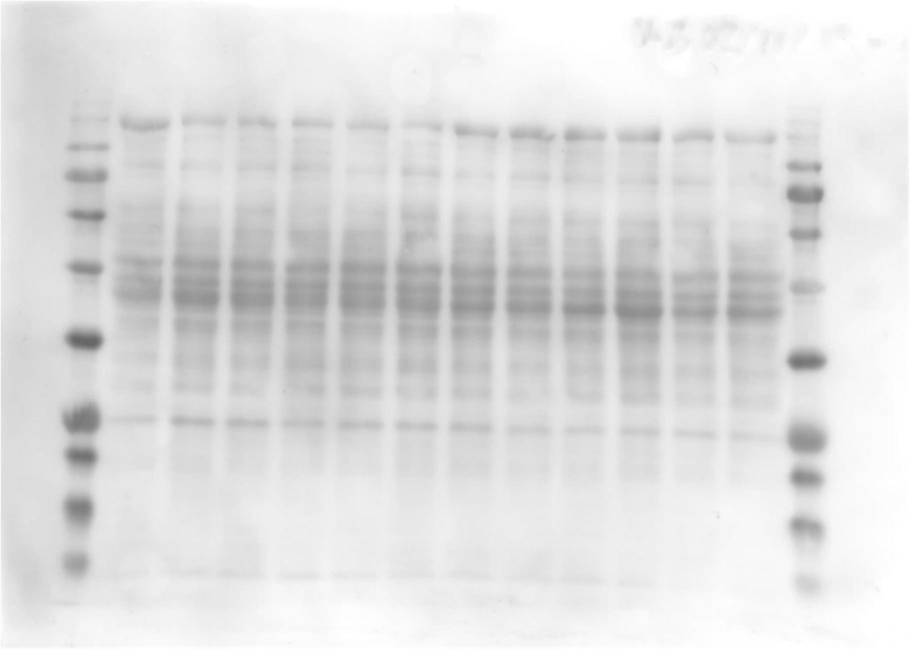

Full unedited gel for Supplemental Figure S1

ARMCX3

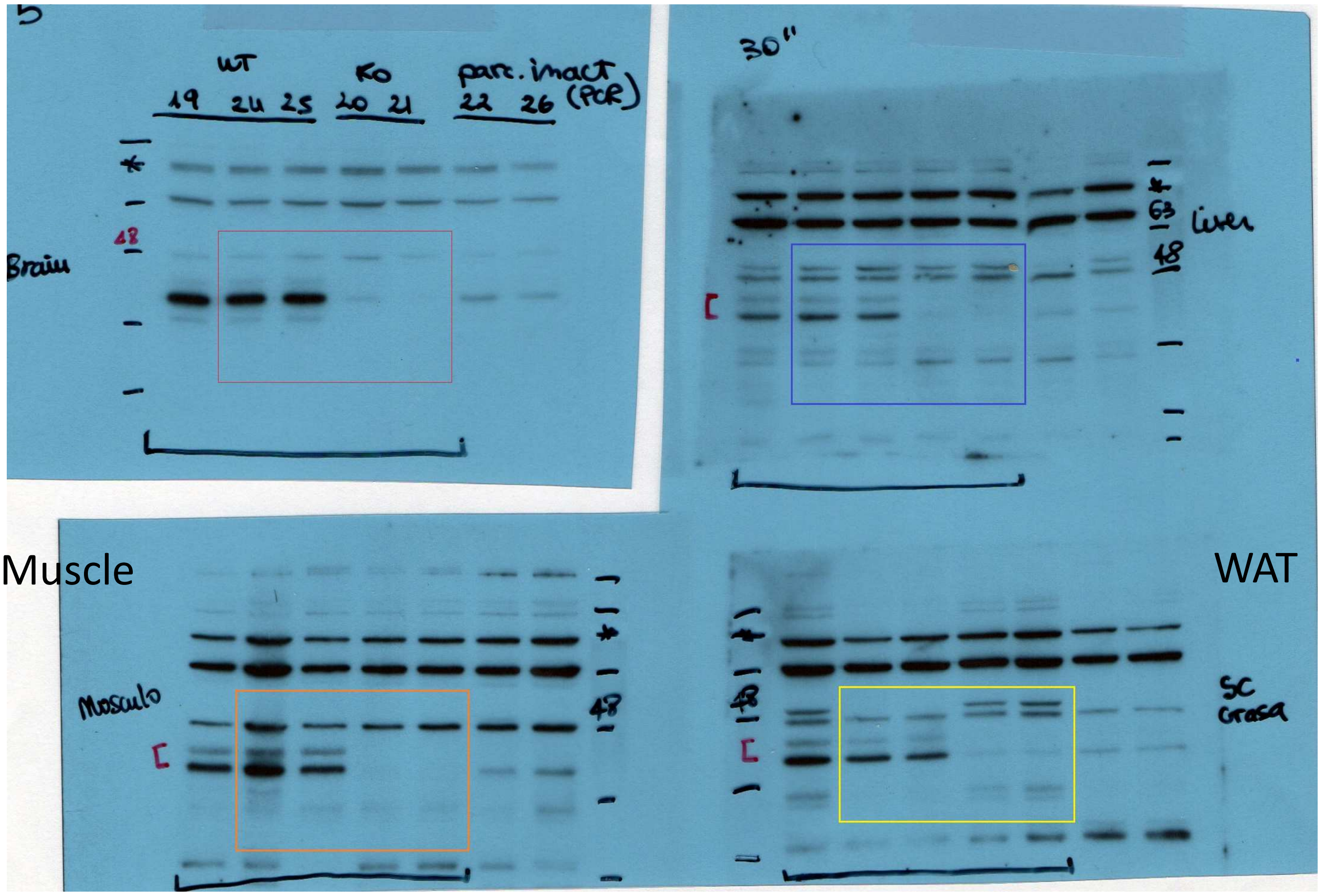

Actin

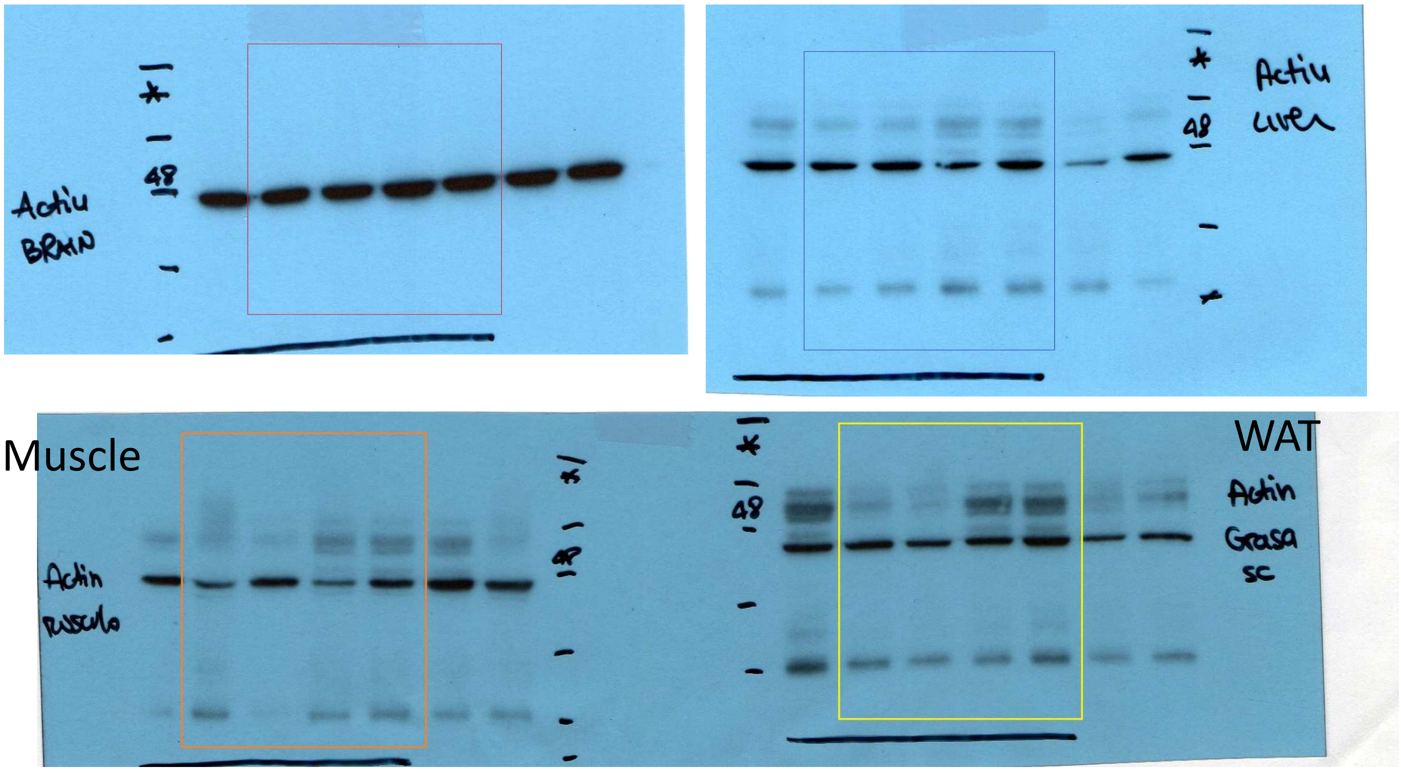

Full unedited gels for Supplemental Figure S6A

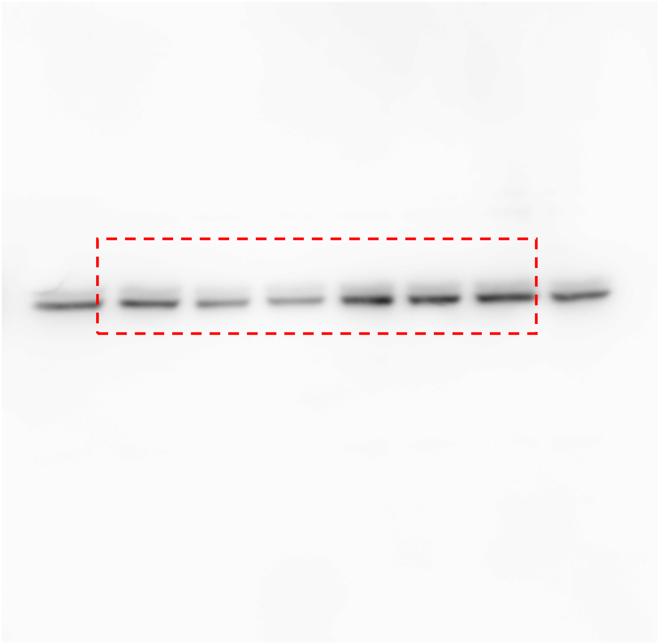

Phospho p44/p42

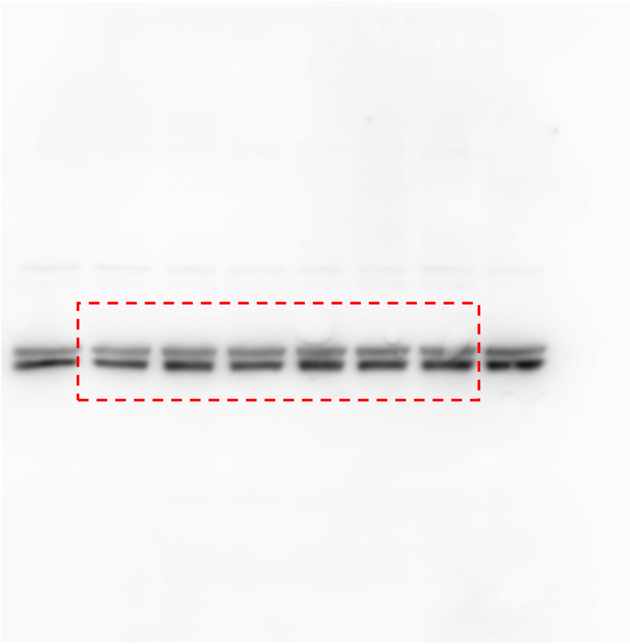

Total p44/p42

Full unedited gels for Supplemental Figure S6A

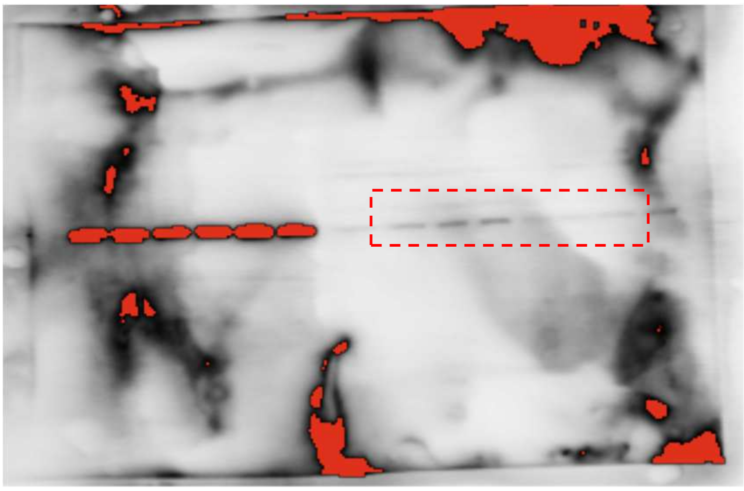

Phospho p38

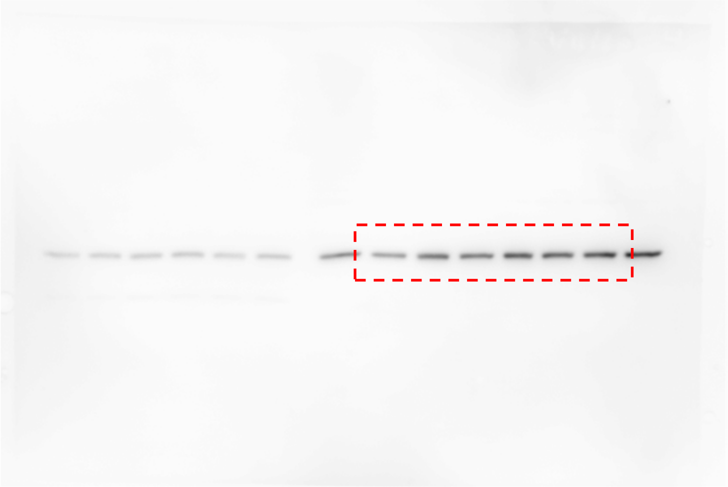

Total p38

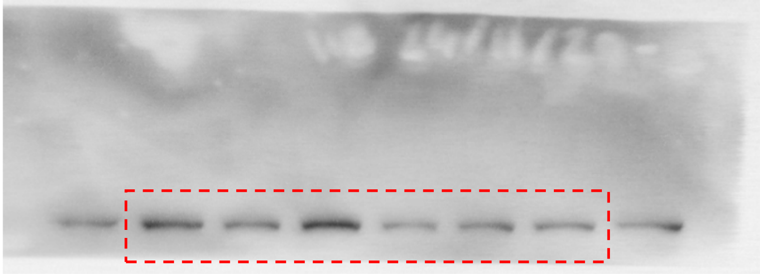

Active β-catenin

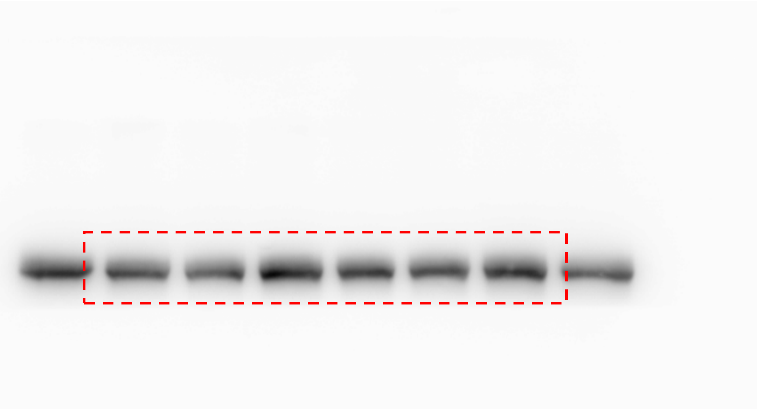

Total β-catenin

Full unedited gels for Supplemental Figure S6B

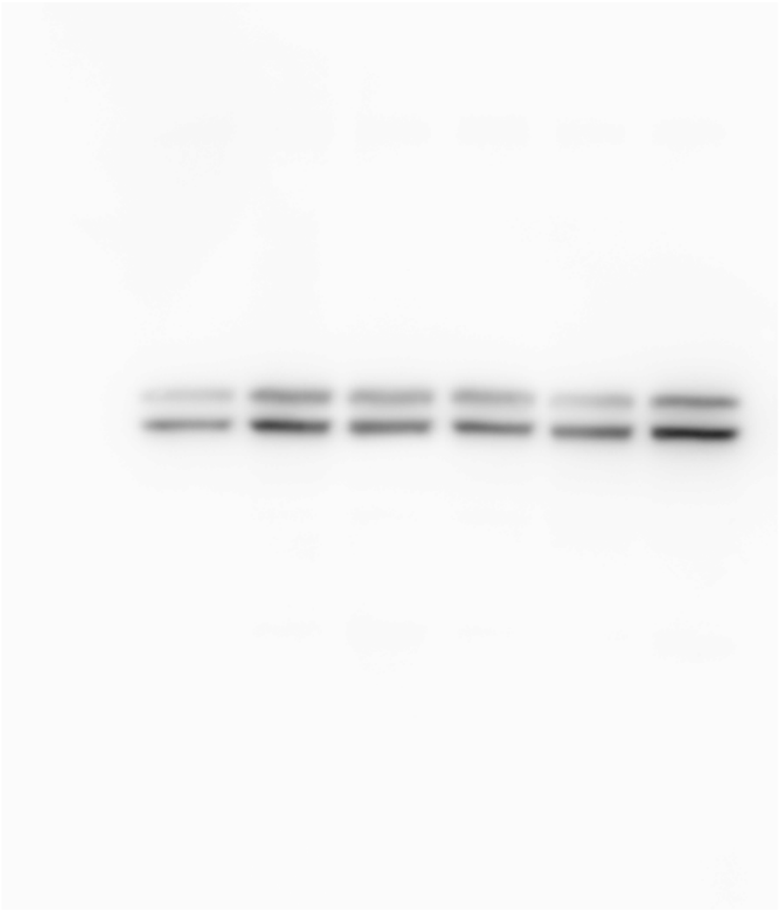

Phospho p44/42

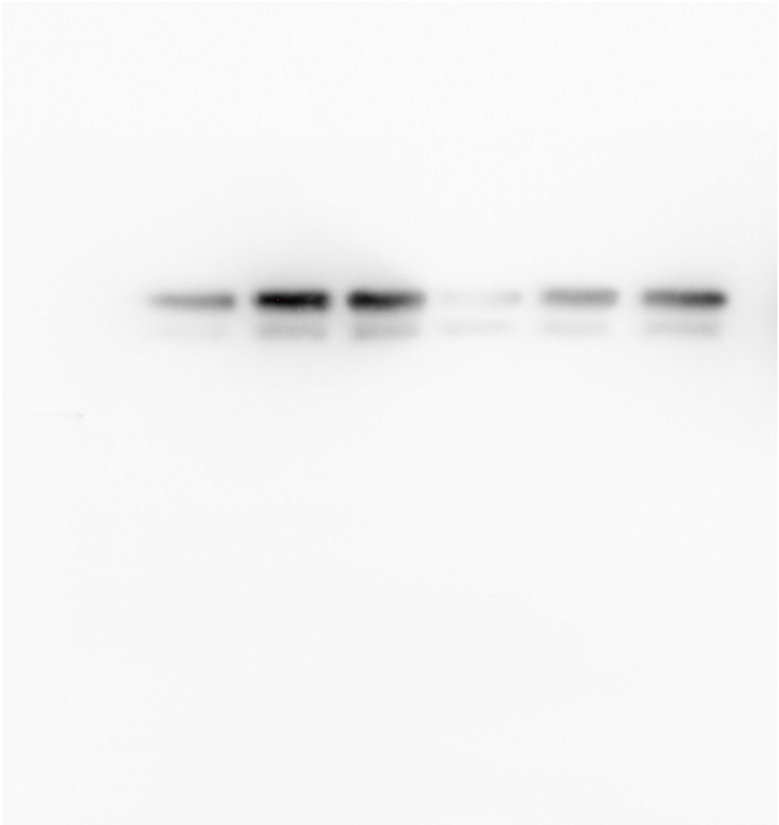

Total p44/42

Full unedited gels for Supplemental Figure S6B

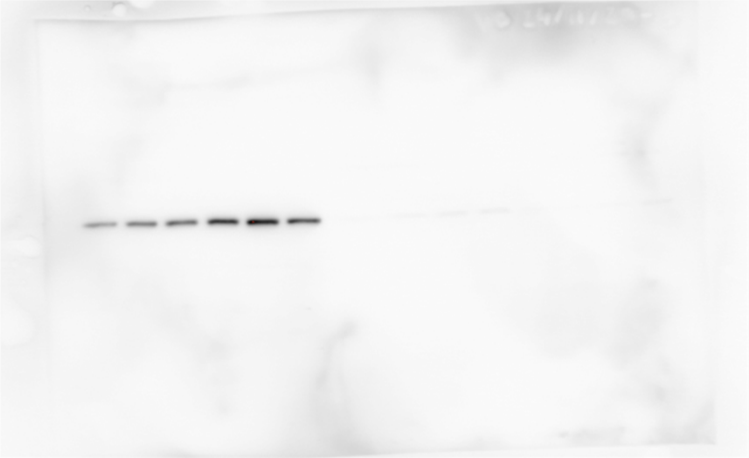

Phospho p38

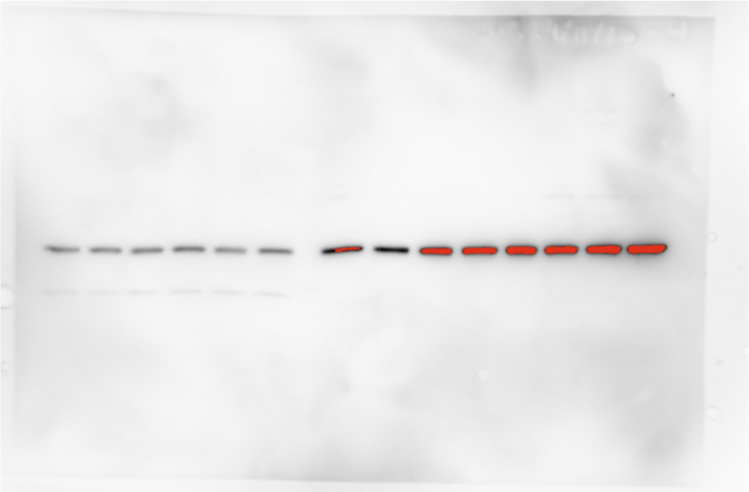

Total p38

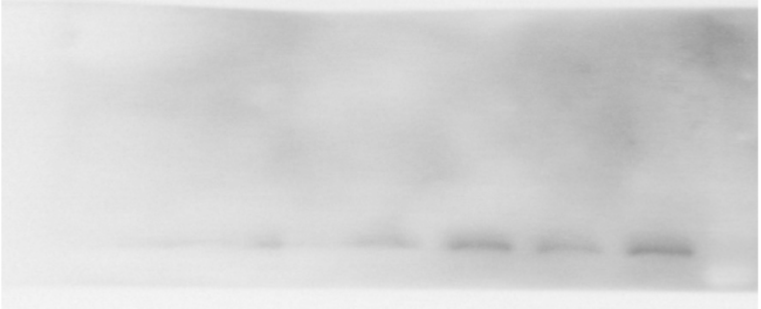

Active β-catenin

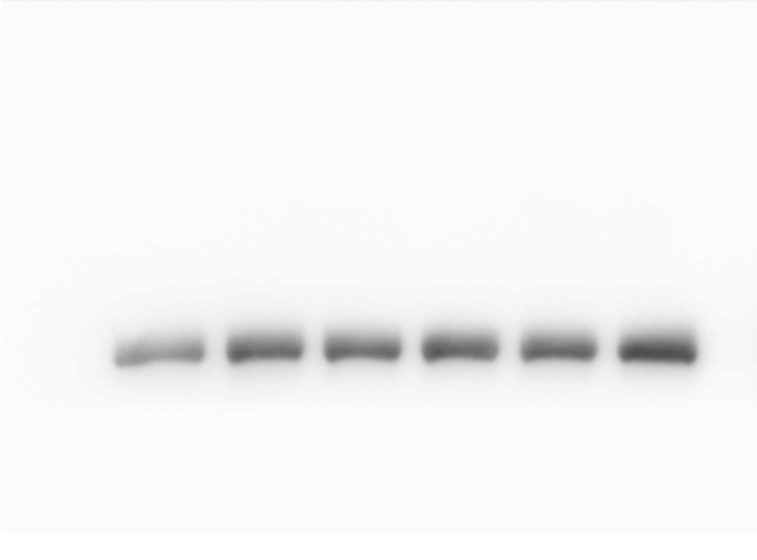

Total β-catenin
